# The association between problematic internet use and social anxiety within adolescents and young adults: a systematic review and meta-analysis

**DOI:** 10.3389/fpubh.2023.1275723

**Published:** 2023-09-29

**Authors:** Haiyang Ding, Bing Cao, Qixuan Sun

**Affiliations:** ^1^Faculty of Psychology, Ministry of Education, Southwest University, Chongqing, China; ^2^Key Laboratory of Cognition and Personality, Faculty of Psychology, Ministry of Education, Southwest University, Chongqing, China; ^3^College of Computer and Information Science, Southwest University, Chongqing, China

**Keywords:** internet addiction disorder, social anxiety, adolescent, young adult, systematic review, meta-analysis

## Abstract

**Objective:**

Although numerous studies have investigated the association between problematic internet use (PIU) and social anxiety, the findings have no yet reached consistent. The present meta-analysis aims to examine the association between PIU and social anxiety within adolescents and young adults (age range: 14–24 years old).

**Method:**

The meta-analysis systematically retrieved the studies prior to September 7, 2023 from Web of Science, PubMed, PsycINFO, Scopus, CNKI, and CQVIP. The meta-analysis based on random-effects model to conduct the research. Stata Version 17.0 and JASP 16.3.0 was used to analysis.

**Results:**

The meta-analysis ultimately included 37 studies (37 effect sizes in total), involving a total of 36,013 subjects. Our findings indicated that the overall correlation between PIU and social anxiety was significant positive [*r* = 0.333, 95% CI (0.292, 0.373), *p* < 0.001]. Their association was significantly moderated by publication year, measurement tools for PIU and social anxiety but not significantly by culture context, developmental level and gender.

**Conclusion:**

This meta-analysis suggests that social anxiety is a predictor of the development of PIU in adolescents and young adults. Furthermore, the study also finds the possibility that contemporary adolescents and youth may exhibit a more “global” behavior pattern, potentially emphasizing fewer differences between cultures, generations and genders.

## 1. Introduction

In light of the progressive development of information technology, an unprecedented increase in internet usage and dependency is observed. Concurrently, there is a significant upswing in the incidence of psychological issues associated with excessive online behavior, known as problematic internet use (PIU) (1). PIU is estimated to affect a noticeable portion of the general population, with a higher prevalence among adolescents and young adults. With studies suggesting that up to 9% of adolescents and young adults are at risk of developing PIU symptoms (2). PIU can lead to the emergence of numerous psychological issues, such as social anxiety. Both these psychological problems and PIU can significantly impact academic performance, social relationships, and overall quality of life for affected adolescents and young adults (3).

Social anxiety in adolescents and young adults can lead to poor academic performance due to avoidance of classroom activities, hinder social interactions (4), elevate the risk of psychological issues like depression, and affect overall psychological well-being (5). Numerous factors can contribute to social anxiety in adolescents and young adults, including genetic predispositions (6), early traumatic events (7), among others. Notably, studies demonstrated that PIU uniquely predicts social anxiety among younger populations, as evidenced by out-of-sample LASSO model cross-validation (8). In addition, research has also substantiated a high comorbidity relationship between PIU and social anxiety within the adolescent and young adult populations (9). This correlation does not extend to adult and older age group. PIU can reduce social skills and intensify feelings of isolation, potentially exacerbating social anxiety symptoms (10).

Several theoretical models have shown that PIU can lead to social anxiety. The cognitive-behavioral model suggests that individuals with social anxiety may resort to social networks or video games as an avoidance strategy, leading to potential PIU (11). The compensatory Internet use theory posits that those with social anxiety use the Internet as a substitute for offline social and emotional connections, which exacerbates social anxiety symptoms and potentially leads to PIU (12). Furthermore, Social anxiety is estimated to affect 7%–13% of the general population, with a higher prevalence among adolescents and young adults. With studies suggesting that up to 15%–20% of college students experience symptoms of social anxiety (13, 14). Although a substantial body of research has established a positive correlation between social anxiety and PIU among adolescents and young adults, there is significant variability in the effect sizes reported across these studies (15–23).

While previous meta-analyses have demonstrated a positive association between PIU and social anxiety, they did not extend their subgroup analyses beyond developmental levels, or the results across different subgroups have not been consistent (24, 25). Differences in societal norms and technological advancements between different time periods or cultural contexts may lead to varying results in studies (24, 25). In addition, the use of different measurement tools may affect the correlation between PIU and social anxiety (26). Previous meta-analysis has also confirmed that the choice of scale can modulate the relationship between social anxiety and PIU (24). Furthermore, while social anxiety and PIU may have distinct manifestations across genders, meta-analytic subgroup effects regarding gender have shown inconsistent results (27, 28). The theory of gender and coping proposes that the way men and women deal with stressors may differ, influencing their vulnerability to developing PIU and social anxiety (29). In an effort to further elucidate the heterogeneity in previous meta-analyses, it is crucial to conduct subgroup analysis. In present study, we consider various factors, including publication year, cultural context, gender, and measurement tools used for PIU and social anxiety.

Although the exponential increase in the number of empirical studies exploring the relationship between PIU and social anxiety among student populations, to the best of our knowledge, no meta-analysis has been conducted to evaluate the overall effect of this relationship within adolescents and young adults. Thus, the current study aims to conduct a meta-analysis to explore the relationship between PIU and social anxiety among adolescents and young adults, with a specific objective to discern whether there are differences compared to other age groups from previous studies. Additionally, we also attempt to explore whether the strength of the relationship between PIU and social anxiety is moderated by effect of subgroups, with the aim of resolving inconsistencies observed in previous meta-analyses regarding subgroup analyses: (a) measurement tools used for PIU, (b) measurement tools used for social anxiety, (c) gender, (d) publication year, and (e) cultural context.

## 2. Materials and method

The current meta-analysis was conducted following the PRISMA (30) guidelines to ensure a rigorous and transparent methodology (see the checklist in Appendix). The PRISMA framework was used to guide the literature search, selection of articles, data extraction, and data synthesis. By adhering to PRISMA, the study aims to enhance the transparency and reliability of the research findings. The protocol of the current meta-analysis has been registered at PROSPERO [ID: CRD42022326313] (31).

### 2.1. Data collection

The present meta-analysis employed a comprehensive approach to identify relevant studies prior to September 7, 2023, utilizing multiple databases including Web of Science, PubMed, PsycINFO, Scopus, CNKI, and CQVIP (CQVIP and CNKI are Chinese databases, and the rest are English databases). Each database was queried using a distinct search formula, as provided in the Appendix. Two researchers independently screened the studies based on inclusion criteria. The collected articles were coded according to author information, year of publication, PIU measurement tool, social anxiety measurement tool country, sample size, male ratio, and age range of subjects.

### 2.2. Inclusion and exclusion criteria

**Figure 1:**
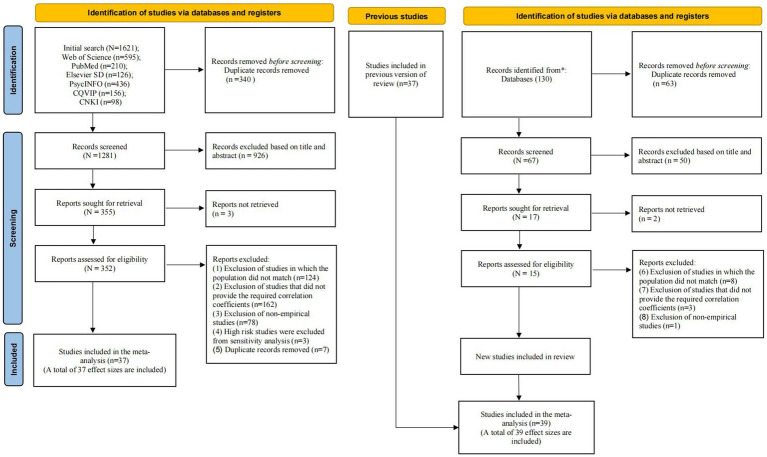
Flow chart process of study selection.

To be eligible for inclusion in this meta-analysis, primary studies had to meet the following PICOS criteria (32): (1) population: studies that involved adolescents and young adults (14–24 years old) as participants, conducted in educational institutions; (2) intervention/exposure: studies that investigated the correlation between PIU and social anxiety using empirical analysis, excluding theoretical studies, review studies, meta-analyses, and case studies; (3) comparison: N/A (4) outcomes: studies that clearly reported sample size and correlation data between variables used in the study; and (5) study design: cross-sectional or longitudinal studies written in Chinese or English. Studies were excluded if they (a) investigated the other kinds of anxiety, (b) had a sample size of less than 30, and (c) were theoretical studies, review studies, meta-analyses, and case studies, (d) targeted on unique student groups such as left-behind children, (e) reported data using only regression analysis, structural equation modeling, and other statistical methods. The selection process yielded 39 relevant studies that met the inclusion criteria and were included in the meta-analysis. See Appendix for the characteristics of included studies. The PRISMA flow chart of the systematic search is depicted in Figure 1.

### 2.3. Study coding and quality assessment

The coding criteria for the studies included in this meta-analysis were divided into two parts: the first part was independent coding of the effect sizes of PIU and social anxiety, and the second part was coding for the correlation of two keywords. The study of pertinent subgroups, such as the respondents’ level of education, cultural background, gender, and measurement methods, was also included in the meta-analysis. The publication year was taken from the publication time of the article, the gender was coded according to the male ratio, and the measurement tools were coded according to the scale used. Cultural classifications are determined based on the dominant culture of the study’s sample. The developmental stage is categorized into youth and adolescents, depending on whether the sample participants are adults (18–24 years old) or not (14–18 years old). To ensure the accuracy of the coding, 2 researchers coded the studies successively with an interval of more than 30 days between the two coding sessions, and the Kappa coefficient was tested to be 0.866, indicating the accuracy of the coding. However, in some cases, there were inconsistencies between the two coders. To resolve these discrepancies, the researchers have consulted with each other and a third-party was consulted to reach a consensus. The meta-analysis utilized *the quality assessment tool for observational cohort and cross-sectional studies* (33) for assessing the studies (Shown in Appendix). The use of this tool enabled a comprehensive evaluation of the included studies, thereby ensuring the rigor and validity of our findings.

### 2.4. Calculation of effect size

In meta-analysis, we often encounter situations where it is necessary to combine correlation coefficients from individual studies into an overall effect size. However, directly combining correlation coefficients poses two major challenges (34). Firstly, correlation coefficients do not follow a normal distribution and their distribution shape varies with the magnitude of the coefficient. Secondly, the variance of r coefficients is not constant but depends on their magnitude.

In the present study, prior to conducting meta-analysis using Stata 17.0 software, the extracted data were subjected to the following transformation according to the formula (34):

a.Fisher’sZ=0.5×ln1+r1−r

b.vz=1n−3

$$c.\;SEz=vz^{0.5}$$

d.Summaryr=e2z−1e2z+1

### 2.5. Data processing

**Figure 2:**
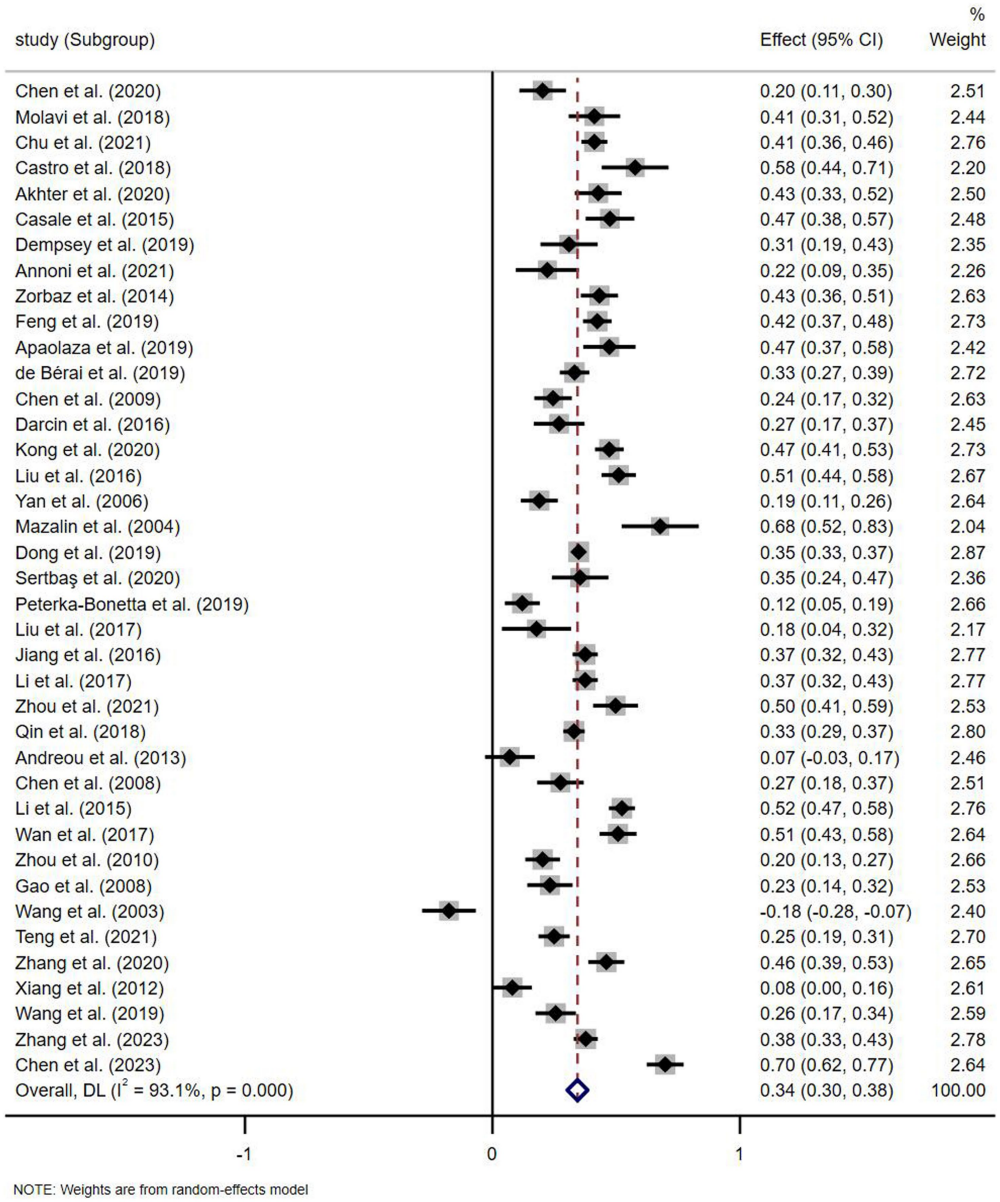
Forest plot of the association PIU and social anxiety.

Random-effects model is a common way to combine effect values. The random effects model assumes that the actual effects may differ across studies and that the different results are affected not only by random errors but also by different samples (35). In this study, we concluded that factors such as the year the study was conducted, the measurement tools for PIU and social anxiety may affect the relationship between problematic Internet use and social anxiety, and thus chose to combine the correlation coefficients in a random effects model. In addition, the test of heterogeneity will be used to determine the need for subgroup analyses and meta-regression, mainly by looking at the significance of the *Q*-test results and the *I*^2^ value, and if the *Q*-test results are significant or the *I*^2^ value is above 75%, the cause of heterogeneity should be explored as much as possible (36). The meta-analysis used the correlation coefficient *r* as an effect value, and Stata 17.0 as well as JASP 16.3 were used to pool effect values and analyze moderating effects. Publication bias is the preference for positive results, resulting in more positive results seen in publications (37), and was assessed in this study using a combination of funnel plots, Egger’s regression coefficient test, and Begger’s rank correlation test. The study also performed a sensitivity analysis (see Figure 2).

## 3. Results

### 3.1. Basic characteristics of included studies

The meta-analysis ultimately included 39 studies (39 effect sizes in total), involving a total of 38,333 subjects, spanning the years 2003 to 2023. The research samples included in the meta-analysis are from China, Iran, Colombia, Bangladesh, Italy, the United States, Switzerland, Turkey, Spain, France, Australia, and Germany. The age range of the participants was from 14 to 24 years old, and there was a total of 16,680 male participants. Basic information of the original studies included in the analysis were shown in Table 1.

**Table 1:** Basic information of the studies included in the meta-analysis.

1st author	Year	*N*	DL	*r*	Measurement (SA)	Measurement (PIU)	Nation	Male
Chen (18)	2020	437	YA	0.200	SPS	SNWAS	China	30%
Molavi (38)	2018	358	YA	0.390	SPI	IAT	Iran	44%
Chu (21)	2021	1,401	YA	0.390	SIAS	SNSATS	China	58%
Casale (39)	2018	214	YA	0.520	SIAS	IAT	Colombia	36%
Akhter (15)	2020	432	YA	0.403	SIAS	GPIUS2	Bangladesh	58%
Casale (39)	2015	400	YA	0.442	SIAS	GPIUS2	Italy	48%
Dempsey (20)	2019	291	YA	0.300	SIAS	FAS (Bergen)	USA	42%
Annoni (3)	2021	240	YA	0.218	SAS	SAS-SV	Switzerland	50%
Zorbaz (40)	2014	682	A	0.407	SAS	PIUSA	Turkey	48%
Feng (23)	2019	1,152	A	0.400	SAS	IAS	China	70%
Apaolaza (41)	2019	346	YA	0.440	SAS	CBS	Spain	48%
de Bérail (42)	2019	1,077	YA	0.320	SAS	IAT	France	27%
Chen (43)	2009	671	YA	0.240	SAS	CIAS	China	52%
Darcin (19)	2016	367	YA	0.142	SAS	BSPS	Turkey	38%
Kong (44)	2020	1,141	A	0.440	SAS	APMPUSQ	China	47%
Liu (45)	2016	800	YA	0.470	SAS	IRDI	China	49%
Biao-Bin (4)	2006	692	A	0.187	SAS	IAT	China	46%
Mazalin (46)	2004	161	YA	0.590	LSAS	IMS	Australia	58%
Dong (47)	2019	10,158	YA	0.335	LSAS	IAT	China	46%
Sertbaş (48)	2020	297	YA	0.340	LSAS	IAS	Turkey	50%
Peterka-Bonetta (49)	2019	773	YA	0.120	IAS	SPAI	German	39%
Liu (45)	2017	200	YA	0.176	IAS	MPATS	China	36%
Jiang (50)	2016	1,488	YA	0.358	IAS	MPATS	China	37%
Li (51)	2017	1,488	YA	0.358	IAS	MPATS	China	37%
Zhou (52)	2011	468	YA	0.460	IAS	MPATS	China	56%
Qin (53)	2018	2056	A	0.318	IAS	IRDI	China	34%
Andreou (17)	2013	384	A	0.070	IAS	IAT	Greek	46%
Chen (54)	2008	437	YA	0.268	IAS	IAT	China	54%
Li (55)	2015	1,380	A	0.48	IAS	IAT	China	52%
Wan (56)	2017	695	YA	0.468	IAS	IAT	China	44%
Zhou (57)	2010	787	YA	0.2	IAS	IAT	China	30%
Gao (58)	2008	461	YA	0.228	IAS	IAT	China	46%
Wang (28)	2003	329	YA	0.174	IAS	IAT	China	51%
Teng (59)	2021	970	YA	0.244	IAS	FAS (Bergen)	China	26%
Zhang (60)	2020	725	YA	0.43	IAS	IAT	China	39%
Xiang (61)	2012	613	YA	0.08	IAS	IAT	China	61%
Wang (62)	2019	578	A	0.250	SAS	MPATS	China	57%
Zhang (63)	2023	1,626	A	0.36	DASS-21	SAS-SV	China	31%
Chen (64)	2023	694	A	0.603	SAD	MPATS	China	50%

DL, developmental level; YA, young adults; A, adolescents; SPS, social phobia scale; SNWAS, social network use and anxiety scale; SIAS, social interaction anxiety scale; SNSATS, social network site addiction scale; GPIUS2, generalized problematic internet use scale 2; FAS (Bergen), Bergen Facebook addiction scale; SAS-SV, social anxiety scale for adolescents-short version; PIUSA, problematic internet use scale for adolescents; CBS, cyber bullying scale; BSPS, brief social phobia scale; APMPUSQ, adolescent pathological mobile phone use scale questionnaire; IRDI, internet-related distress scale; LSAS, Liebowitz social anxiety scale; IMS, Internet-motivation scale; IAS, internet addiction scale; SPAI, social phobia and anxiety inventory; MPATS, mobile phone addiction tendency scale; IMS, internet-motivation scale; SPI, social phobia inventory; DASS-21, depression, anxiety, stress scales-21; SAD, social avoidance and distress scale; SA, social anxiety.

### 3.2. Heterogeneity analysis

The results of the heterogeneity test revealed that the *Q* test for the effect value of the relationship between problematic network use and social anxiety was significant, with a *Q* value of 553.55 (*p* < 0.001) and a value of 93.1% for *I*^2^, which exceeded the 75% rule (36), indicating that the results were heterogeneous.

### 3.3. Main effect estimation

The results showed that the overall correlation between PIU and social anxiety was 0.344 (*z* = 16.384, *p* < 0.001) with a 95% CI of (0.302, 0.385), as determined by Fisher’s *Z* transformation of the correlation coefficients. According to the classification criteria for the size of the correlation, the correlation between the two was relatively strong and varied between 0.10 and 0.40 (65).

### 3.4. Subgroup analysis and meta-regression results

According to the results of the heterogeneity test, the random effects model was used to test the moderating effects of categorical variables, and the moderating effects of PIU measurement tools, social anxiety measurement tools and subjects’ gender, cultural background and developmental level were analyzed, and the results are shown in Table 2. We also conducted the subgroup analyses with the type of databases (Chinese/English; shown in Appendix).

**Table 2:** Results of subgroup analysis.

Subgroup	Heterogeneity		Type	*k*	Effect size and 95% interval			Test of null (2-tail)	
	*Q*_B_	*df*			*r*	LL	UL	*Z*	*p*
PIU measurement	20.97^***^	4	IAT	17	0.298	0.230	0.367	8.547	<0.001
			MPATS	5	0.346	0.269	0.424	8.760	<0.001
			GPIUS2	2	0.450	0.382	0.518	12.935	<0.001
			FAS	2	0.263	0.209	0.318	9.314	<0.001
			Others	13	0.398	0.319	0.476	9.893	<0.001
Social anxiety measurement	10.81^*^	4	SIAS	5	0.433	0.368	0.499	12.886	<0.001
			SAS	11	0.350	0.283	0.418	12.886	<0.001
			IAS	16	0.272	0.193	0.352	6.708	<0.001
			LSAS	3	0.444	0.284	0.604	5.446	<0.001
			Others	4	0.423	0.231	0.616	4.308	0.003
Developmental level	0.04	1	Adolescents	8	0.333	0.292	0.373	7.277	<0.001
			Young adults	31	0.330	0.284	0.376	14.006	<0.001
Culture	0.17	1	Eastern	26	0.338	0.289	0.386	13.610	<0.001
			Western	13	0.358	0.273	0.444	8.213	<0.001
Gender	0.04	1	M > F	14	0.351	0.246	0.455	6.586	<0.001
			F > M	25	0.340	0.300	0.380	16.384	<0.001

PIU, problematic internet use; M, male; F, female. IAT, internet addiction test; MPATS, mobile phone addiction tendency scale; GPIUS2, generalized problematic internet use scale 2; FAS, Facebook addiction scale; SIAS, social interaction anxiety scale; LSAS, Liebowitz social anxiety scale.

Meta-regression was conducted on publication year to investigate the sources of heterogeneity and publication year could explain the heterogeneity of meta-analysis [*t* = 2.09*, p* = 0.044 < 0.05; 95% CI (0.004.0.281); shown in Table 3].

**Table 3:** Meta-regression of publication year.

_ES	Coefficient	Std. err.	*t*	*p*	95% CI
Year	0.014	0.006	2.09	0.044	(0.004, 0.281)
_cons	−28.397	13.7567	−2.06	0.046	(−56.271, −0.523)

Year, publication year.

### 3.5. Publication bias test

**Figure 3:**
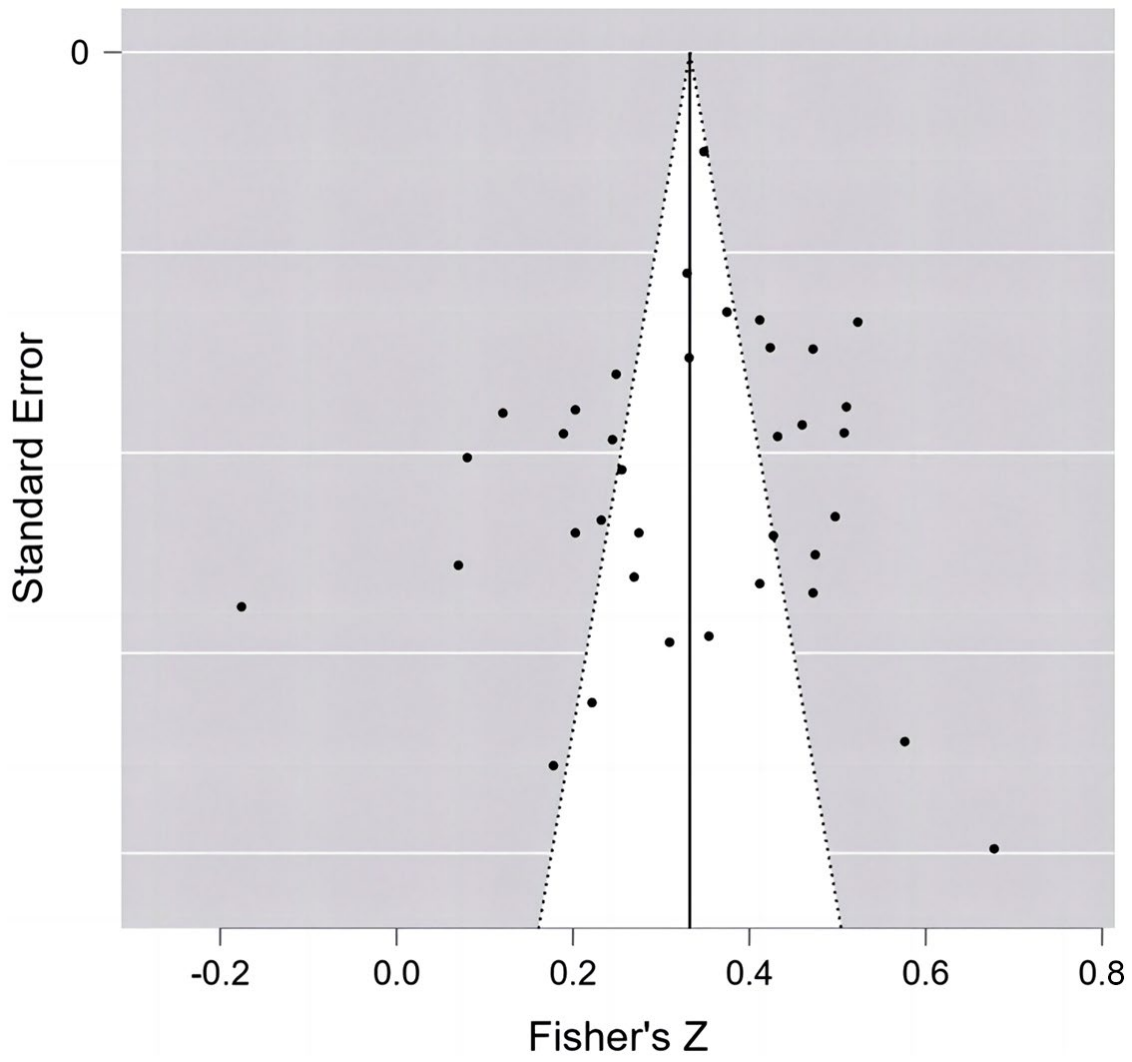
Funnel plot of the association PIU and social anxiety.

In testing for publication bias, the results were first examined by means of a funnel plot. As seen in Figure 3, the studies are more evenly distributed, which can out not indicate that studies targeting the relationship between the two may not have publication variance. For further publication bias testing, Egger’s regression coefficient test with fail-safe *N* test was used.

Publication bias is less likely if fail-safe *N* is greater than 5*K* + 10 (*K* represents the number of independent samples) (37). Fail-safe *N* results showed that *N* = 42,768 > 5*K* + 10. The results of the Egger regression coefficients showed that the intercept of the social anxiety regression equation did not reach a significant level (*z* = −0.235, *p* = 0.814 > 0.05) indicating that there was no significant publication bias in the current study. In conclusion, there was no significant publication bias in the current meta-analysis.

### 3.6. Sensitivity analysis

The meta-analysis tested several potential changes, including excluding certain studies, using different statistical methods, and evaluating potential publication bias, and the results consistently showed that the main conclusions remained unchanged. Therefore, we conclude that the meta-analysis results in this study are highly reliable and robust, suitable for informing decision-making and clinical practice in this field. The sensitive analysis table can be seen in the Appendix.

## 4. Discussion

The present meta-analysis revealed a significant positive correlation between PIU and social anxiety. The study also advances the current understanding of the relationship between PIU and social anxiety is moderated by effect of subgroups: measurement tools, publication year. Specifically, we found that publication year does in fact explain some of the heterogeneity observed across studies while previous meta-analysis have indicated that publication year does not moderate the relationship between PIU and social anxiety (25). The findings contribute to a more nuanced and comprehensive understanding of the association between PIU and social anxiety.

### 4.1. Overall association between PIU and social anxiety

This study employed a meta-analytic methodology to investigate the association between PIU and social anxiety within the adolescent and young adult sample. The meta-analysis revealed a robust and positive correlation between PIU and social anxiety. The results suggest that individuals with elevated levels of PIU are more likely to report greater levels of social anxiety. The compensatory Internet use theory suggests that individuals with social anxiety may treat Internet as a “compensatory” mechanism for their lack of social and emotional connections in the offline world, which can lead to dependence on the Internet for social interactions, exacerbating symptoms of social anxiety and leading to PIU (12). Therefore, PIU has the potential to exert a detrimental influence on the social and emotional well-being of students, which in turn may culminate in academic obstacles.

In addition, among the 39 studies included in this meta-analysis, only one study reported a significant negative correlation between PIU and social anxiety among adolescents and young adults (28). Notably, the number of participants with PIU in that study was significantly less than that of similar studies conducted. This result may be attributed to several factors. First, the issue of sampling bias must be considered, as some studies were conducted online, and in such cases, individuals with a greater interest in Internet use may be more likely to participate. In contrast, the aforementioned study was conducted offline and limited to schools with restricted Internet access. Second, the study’s age was relatively dated, and people spent less time online than today.

### 4.2. Heterogeneity with subgroups

The present meta-analysis utilized subgroup analysis to explore the potential effects of publication year, measurement tools for PIU and social anxiety, cultural background, and gender on the association between PIU and social anxiety. The findings revealed that while the subgroup analysis of measurement tools for PIU and social anxiety and publication year demonstrated a significant effect, the subgroup analysis of cultural background, and gender did not yield significant effects.

#### 4.2.1. Meta-regression analysis of publication year

The current meta-analysis indicated a significant meta-regression effect of publication year on the correlation between PIU and social anxiety among adolescents and young adults while previous meta-analysis found that early studies on PIU and social anxiety may have had a biased sample leading to publication years not explaining heterogeneity (25). Specifically, the strength of the correlation has increased over time. This finding observed variation could be attributed to a multitude of factors. These may include the advent of new assessment tools for PIU and social anxiety, heightened identification of at-risk populations, alterations in Internet activities, and advancements in accessibility and technology of online platforms, among others.

#### 4.2.2. Subgroup analysis of measurement tools for PIU and social anxiety

The finding regarding the moderating effect of measurement tools on the relationship between PIU and social anxiety highlights the importance of careful tool selection in research, this was inconsistent with the findings of previous meta-analyses (24, 66). One plausible explanation for these inconsistencies lies in the ongoing absence of consensus regarding the precise definitions and criteria for PIU and social anxiety. The lack of consensus around the definition and criteria of PIU and social anxiety has resulted in no universally accepted measurement tool, may leading to inconsistencies in findings (67, 68).

#### 4.2.3. Subgroup analysis of cultural context

**Figure 4:**
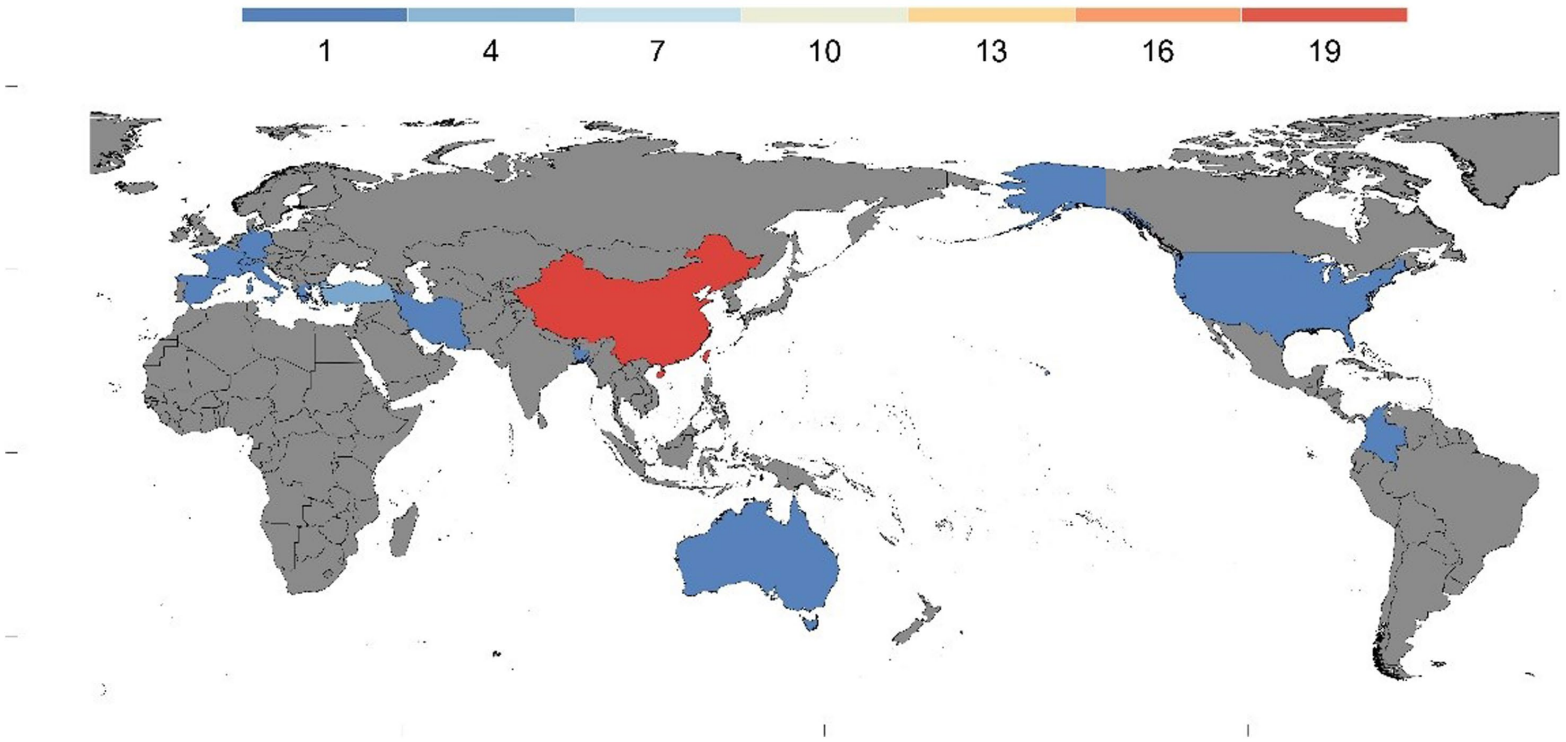
Sample distribution map.

Through subgroup analysis, we found that the cultural context may contribute to reduced heterogeneity of the sample. While previous research has suggested that cultural background may moderate the relationship between PIU and social anxiety within adult sample (24), the present study did not find a significant moderating effect of cultural background. One possible explanation for this discrepancy is the difference in the sample populations used in present studies. As Figure 4 shows, the present study mainly focused on a sample of students from a single cultural background (Chinese). Current research may suggest that contemporary adolescents and youth exhibit more “global” characteristics, indicating that cultural differences may be less pronounced than they were before. The lack of significant moderating effects of cultural background in the present study suggests that the relationship between PIU and social anxiety may be relatively stable across different cultural contexts.

#### 4.2.4. Subgroup analysis of gender

We also found that the gender may contribute to reduced heterogeneity of the sample through subgroup analysis. Previous research has suggested that there may be differences in the preferences of males and females for gaming and social applications, which could affect their use of mobile devices and their risk of potential addictive behaviors. For example, a study found that males were more likely to use game applications with competitive and adventurous characteristics, while females were more likely to use social applications (69). However, many other studies have not found a direct relationship between gender and PIU (70, 71). This suggests that while males and females may have different preferences and behaviors, gender itself is not a key factor influencing the relationship between PIU and social anxiety.

#### 4.2.5. Subgroup analysis of developmental level

We found no significant difference between adolescents and youth in the relationship between PIU and social anxiety. This may be due to the pervasive use of internet across these age groups, and the relatively similar social contexts they are embedded in, such as school or university environments where Internet use is prevalent and often necessary for both academic and social purposes. It is possible that the similar exposure to online environments and the comparable pressures they face in these stages of their lives lead to no significant variance in the PIU-social anxiety relationship across these groups.

Previous research has revealed a significant difference between adolescents and the adult group (including middle-aged and older individuals) (25). This could be attributed to the fact that adults, particularly those in middle and older age, may have different internet usage habits compared to younger individuals. Adults may use the Internet more for practical purposes such as work, information seeking or maintaining social connections, rather than for leisure or as a primary social outlet. Moreover, the level of digital literacy and the role of the Internet in daily life can also differ significantly between these age groups, which can contribute to the differential impacts of PIU on social anxiety.

## 5. Limitations and prospects

The principal merits of the meta-analysis are its revelation of the association between PIU and social anxiety in the adolescent and young adult population and the meta-analysis has also identified heterogeneous explanatory factors that were not previously reported in the literature, while also providing novel insights for cross-cultural research in this field. Nevertheless, the study possesses several limitations. For starters, the prime demerit is undeniably that the predominantly cross-sectional nature of the literature, limiting our ability to infer causality. Longitudinal designs would allow researchers to identify whether PIU precedes social anxiety, or if social anxiety leads to PIU, or if the relationship is bi-directional. Understanding these dynamics could be crucial for developing effective preventative measures and interventions. The second noteworthy demerit is the method of conducting a survey, The majority of the studies included in our meta-analysis collected data through online questionnaires. A potential limitation of this method lies in the self-selection bias inherent to online research. Future research should aim to address this limitation by adopting more diverse data collection methods. For instance, offline methods such as in-person interviews or paper-and-pencil questionnaires can be used to include individuals who might be less inclined to participate in online research.

Given that college students comprise the primary study subjects in the field, subsequent research in the field should include more representative sampling methods, such as stratified sampling or random sampling, can be employed to ensure the inclusion of diverse demographic groups, including individuals with varying levels of internet use and interest.

## 6. Conclusion

The meta-analysis utilized the random effects model to quantitatively analyze the association between PIU and social anxiety among adolescents and young adults (age range: 14–24 years old). The results revealed a significant positive correlation between PIU and social anxiety, indicating that social anxiety is a predictor of PIU development in this age group. Subgroup analysis and meta-regression results identified significant differences in the relationship between PIU and social anxiety based on the publication year and measurement tools used. However, no significant differences were found with regards to developmental level, gender or cultural context.

## Author contributions

HD: Conceptualization, Data curation, Formal analysis, Investigation, Methodology, Project administration, Resources, Software, Validation, Writing – original draft, Writing – review & editing. BC: Project administration, Software, Writing – review & editing. QS: Visualization, Writing – review & editing.
