# Supplementary material for: The association between problematic internet use and social anxiety within adolescents and young adults: a systematic review and meta-analysis
Source: Front Public Health. 2023 Sep 29;11:1275723. doi: 10.3389/fpubh.2023.1275723 (PMC10570444; doi:10.3389/fpubh.2023.1275723)

Literature included in the meta-analysis (39 studies)

1. Akhter, Md. S., & Khalek, Md. A. (2020). Association Between Psychological Well-Being and Problematic Internet Use Among University Students of Bangladesh. Journal of Technology in Behavioral Science, 5(4), 357–366. https://doi.org/10.1007/s41347-020-00142-x

2. Alexander Castro, J., Vinaccia, S., & Ballester-Arnal, R. (2018). Social anxiety, Internet and Cibersex addiction: its relationship with health perception. Terapia Psicologica, 36(3), 134-143. https://doi.org/10.4067/s0718-48082018000300134

3. Andreou, E., & Svoli, H. (2013). The association between internet user characteristics and dimensions of internet addiction among Greek adolescents. International Journal of Mental Health and Addiction, 11(2), 139-148. https://doi.org/http://dx.doi.org/10.1007/s11469-012-9404-3

4. Annoni, A. M., Petrocchi, S., Camerini, A.-L., & Marciano, L. (2021). The Relationship between Social Anxiety, Smartphone Use, Dispositional Trust, and Problematic Smartphone Use: A Moderated Mediation Model. International Journal of Environmental Research and Public Health, 18(5), 2452. https://doi.org/10.3390/ijerph18052452

5. Apaolaza, V., Hartmann, P., D'Souza, C., & Gilsanz, A. (2019). Mindfulness, compulsive mobile social media use, and derived stress: The mediating roles of self-esteem and social anxiety. Cyberpsychology, Behavior, and Social Networking, 22(6), 388-396. https://doi.org/http://dx.doi.org/10.1089/cyber.2018.0681

6. Atroszko, P. A., Balcerowska, J. M., Bereznowski, P., Biernatowska, A., Pallesen, S., & Andreassen, C. S. (2018). Facebook addiction among Polish undergraduate students: Validity of measurement and relationship with personality and well-being. Computers in Human Behavior, 85, 329-338. https://doi.org/http://dx.doi.org/10.1016/j.chb.2018.04.001

7. Casale, S., & Fioravanti, G. (2015). Satisfying needs through Social Networking Sites: A pathway towards problematic Internet use for socially anxious people? Addictive behaviors reports, 1, 34-39. https://doi.org/https://doi.org/10.1016/j.abrep.2015.03.008

8. Chen, D., Zhang, J. M., Shen, L. L. & Liao, Z.. (2009). Internet addiction and its relationship with social anxiety among college students. Chinese Journal of Health Psychology (02), 151-152. doi:10.13342/j.cnki.cjhp.2009.02.029.

9. Chen, Jun, & Fan, Jia-Lu. (2008). Analysis of Internet addiction status and psychological characteristics of medical students. Chinese Family Medicine(11), 963-965.

10. Choi, M., Park, S., & Cha, S. (2017). Relationships of Mental Health and Internet Use in Korean Adolescents. Archives of Psychiatric Nursing, 31(6), 566-571. https://doi.org/10.1016/j.apnu.2017.07.007

11. Chu, X., Ji, S., Wang, X., Yu, J., Chen, Y., & Lei, L. (2021). Peer Phubbing and Social Networking Site Addiction: The Mediating Role of Social Anxiety and the Moderating Role of Family Financial Difficulty. Frontiers in Psychology, 12, Article 670065. https://doi.org/10.3389/fpsyg.2021.670065

12. Darcin, A. E., Kose, S., Noyan, C. O., Nurmedov, S., Yilmaz, O., & Dilbaz, N. (2016). Smartphone addiction and its relationship with social anxiety and loneliness. Behaviour & Information Technology, 35(7), 520-525. https://doi.org/10.1080/0144929x.2016.1158319

13. de Bérail, P., Guillon, M., & Bungener, C. (2019). The relations between YouTube addiction, social anxiety and parasocial relationships with YouTubers: A moderated-mediation model based on a cognitive-behavioral framework. Computers in Human Behavior, 99, 190-204. https://doi.org/http://dx.doi.org/10.1016/j.chb.2019.05.007

14. Dempsey, A. E., O'Brien, K. D., Tiamiyu, M. F., & Elhai, J. D. (2019). Fear of missing out (FoMO) and rumination mediate relations between social anxiety and problematic Facebook use. Addictive behaviors reports, 9, 7. https://doi.org/http://dx.doi.org/10.1016/j.abrep.2018.100150

15. Dong, B., Zhao, F., Wu, X.-S., Wang, W.-J., Li, Y.-F., Zhang, Z.-H., & Sun, Y.-H. (2019). Social anxiety may modify the relationship between internet addiction and its determining factors in Chinese adolescents. International Journal of Mental Health and Addiction, 17(6), 1508-1520. https://doi.org/http://dx.doi.org/10.1007/s11469-018-9912-x

16. Gao Ting. (2008). A study on the relationship between Internet addiction and social anxiety among college students. Journal of Shanxi Youth Management Cadre College, 21(1). http://qikan.cqvip.com/Qikan/Article/Detail?id=26732795

17. Jaiswal, A., Manchanda, S., Gautam, V., Goel, A. D., Aneja, J., & Raghav, P. R. (2020). Burden of internet addiction, social anxiety and social phobia among University students, India. Journal of Family Medicine and Primary Care, 9(7), 3607-3612. https://doi.org/10.4103/jfmpc.jfmpc_360_20

18. Kong, F., Qin, J., Huang, B., Zhang, H., & Lei, L. (2020). The effect of social anxiety on mobile phone dependence among Chinese adolescents: A moderated mediation model. Children and Youth Services Review, 108, Article 104517. https://doi.org/10.1016/j.childyouth.2019.104517

19. Lee, Y. Z.. (2015). The association between social anxiety emotional intelligence and Internet addiction among high school students in Henan. School Health in China, 36(11), 1732-1733+1736. https://doi.org/10.16835/j.cnki.1000-9817.2015.11.047

20. Liu X. (2017). The relationship between cell phone addiction and social anxiety among college students:The mediating role of interpersonal relationships. Journal of Southwest Medical University, 40(4).

21. Mazalin, D., & Moore, S. (2004). Internet use, identity development and social anxiety among young adults. Behaviour Change, 21(2), 90-102. https://doi.org/10.1375/bech.21.2.90.55425

22. Molavi, P., Mikaeili, N., Ghaseminejad, M. A., Kazemi, Z., & Pourdonya, M. (2018). Social Anxiety and Benign and Toxic Online Self-Disclosures: An Investigation Into the Role of Rejection Sensitivity, Self-Regulation, and Internet Addiction in College Students. The Journal of nervous and mental disease, 206(8), 598–605. https://doi.org/10.1097/NMD.0000000000000855

23. Peng S. (2020). The relationship between negative appraisal fear and college students' Internet overuse: The mediating role of social anxiety and self-control. Psychological Science, 0(1). http://qikan.cqvip.com/Qikan/Article/Detail?id=00002EOJL33O7JP0MPDO8JP16LR

24. Peterka-Bonetta, J., Sindermann, C., Elhai, J. D., & Montag, C. (2019). Personality Associations With Smartphone and Internet Use Disorder: A Comparison Study Including Links to Impulsivity and Social Anxiety. Frontiers in Public Health, 7, Article 127. https://doi.org/10.3389/fpubh.2019.00127

25. Qin J.X. (2018). The role of adolescents' social self-efficacy and satisfaction on mobile social network use and interaction anxiety. China School Health, 39(4).

26. Sertbaş, K., Çutuk, S., Soyer, F., Akkuş Çutuk, Z., & Aydoğan, R. (2020). Mediating role of emotion regulation difficulties in the relationship between social anxiety and problematic Internet use. Psihologija, 53(3), 291-305. https://doi.org/http://dx.doi.org/10.2298/PSI190730013S

27. Teng X.C., et al." The effect of social anxiety on social network addiction in college students: the moderating effect of intentional self-regulation." Chinese Journal of Clinical Psychology.03(2021):514-517. doi:10.16128/j.cnki.1005-3611.2021.03.014.

28. Wang, J.-L., Sheng, J.-R., & Wang, H.-Z. (2019). The Association Between Mobile Game Addiction and Depression, Social Anxiety, and Loneliness. Frontiers in Public Health, 7, Article 247. https://doi.org/10.3389/fpubh.2019.00247

29. Wang, L. H., & Dong, F. J. (2003). A study of the relationship between Internet addiction and social support, relationship anxiety, and self-concordance among college students. Journal of Health Psychology (02), 94-96. https://doi.org/10.13342/j.cnki.cjhp.2003.02.006

30. Wan J.J. (2017). A study of individual correlates of online relationship addiction among college students. Green Technology, 0(21). http://qikan.cqvip.com/Qikan/Article/Detail?id=673851590

31. Xiang H, & Ma X.L. (2012). A survey of risk factors for Internet addiction among college students. Modern Preventive Medicine, 39(04), 922-924.

32. Yan Biao-Bin. (2006). A study on the relationship between adolescents' online behavior and social development. Applied Psychology, 12(2). http://qikan.cqvip.com/Qikan/Article/Detail?id=22756034

33. Yuan X.X. (2007). A study on the psychology of Internet addiction among college students. Journal of Ningbo University: Education Science Edition, 29(3). http://qikan.cqvip.com/Qikan/Article/Detail?id=24716910

34. Zhang, G. C., & Chen, M.. (2020). The effect of self-control on college students' Internet addiction: a mediating model of regulation. Chinese Journal of Health Psychology, 28(07), 1090-1095. https://doi.org/10.13342/j.cnki.cjhp.2020.07.030

35. Zhou Y.Q. (2010). Analysis of internet addiction status and influencing factors among college students. Chinese Family Medicine, 13(31). http://qikan.cqvip.com/Qikan/Article/Detail?id=35866723

36. Zhou, Yuan-Chun, & Jin, Wen-Mei. (2011). A survey of Internet addiction among college students: A case study of Guangdong Technical Teachers' College. Journal of Guangdong Technical Teachers' College, 32(06), 82-84.

37. Zorbaz, O., & Tuzgol Dost, M. (2014). Examination of Problematic Internet Use of High School Student in Terms of Gender, Social Anxiety and Peer Relations. Hacettepe Universitesi Egitim Fakultesi Dergisi-Hacettepe University Journal of Education, 29(1), 298-310. ://WOS:000334326600022

38. Zhang L, Wang B, Xu Q and Fu C (2023) The role of boredom proneness and self-control in the association between anxiety and smartphone addiction among college students: a multiple mediation model. Front. Public Health 11:1201079. doi: 10.3389/fpubh.2023.1201079

39. Chen C, Shen Y, Lv S, Wang B and Zhu Y (2023) The relationship between self-esteem and mobile phone addiction among college students: The chain mediating effects of social avoidance and peer relationships. Front. Psychol. 14:1137220. doi: 10.3389/fpsyg.2023.1137220

Supplementary Table 1. PRISMA 2020 Checklist

Section and Topic	Item #	Checklist item
**TITLE**		
Title	1	Identify the report as a systematic review.
**ABSTRACT**		
Abstract	2	See the PRISMA 2020 for Abstracts checklist.
**INTRODUCTION**		

Section and Topic	Item #	Checklist item
Rationale	3	Describe the rationale for the review in the context of existing knowledge.
Objectives	4	Provide an explicit statement of the objective(s) or question(s) the review addresses.
**METHODS**		
Eligibility criteria	5	Specify the inclusion and exclusion criteria for the review and how studies were grouped for the syntheses.
Information sources	6	Specify all databases, registers, websites, organisations, reference lists and other sources searched or consulted to identify studies. Specify the date when each source was last searched or consulted.
Search strategy	7	Present the full search strategies for all databases, registers and websites, including any filters and limits used.
Selection process	8	Specify the methods used to decide whether a study met the inclusion criteria of the review, including how many reviewers screened each record and each report retrieved, whether they worked independently, and if applicable, details of automation tools used in the process.
Data collection process	9	Specify the methods used to collect data from reports, including how many reviewers collected data from each report, whether they worked independently, any processes for obtaining or confirming data from study investigators, and if applicable, details of automation tools used in the process.
Data items	10a	List and define all outcomes for which data were sought. Specify whether all results that were compatible with each outcome domain in each study were sought (e.g. for all measures, time points, analyses), and if not, the methods used to decide which results to collect.
	10b	List and define all other variables for which data were sought (e.g. participant and intervention characteristics, funding sources). Describe any assumptions made about any missing or unclear information.
Study risk of bias assessment	11	Specify the methods used to assess risk of bias in the included studies, including details of the tool(s) used, how many reviewers assessed each study and whether they worked independently, and if applicable, details of automation tools used in the process.
Effect measures	12	Specify for each outcome the effect measure(s) (e.g. risk ratio, mean difference) used in the synthesis or presentation of results.
Synthesis methods	13a	Describe the processes used to decide which studies were eligible for each synthesis (e.g. tabulating the study intervention characteristics and comparing against the planned groups for each synthesis (item #5)).
	13b	Describe any methods required to prepare the data for presentation or synthesis, such as handling of missing summary statistics, or data

Section and Topic	Item #	Checklist item
		conversions.
	13c	Describe any methods used to tabulate or visually display results of individual studies and syntheses.
	13d	Describe any methods used to synthesize results and provide a rationale for the choice(s). If meta-analysis was performed, describe the model(s), method(s) to identify the presence and extent of statistical heterogeneity, and software package(s) used.
	13e	Describe any methods used to explore possible causes of heterogeneity among study results (e.g. subgroup analysis, meta-regression).
	13f	Describe any sensitivity analyses conducted to assess robustness of the synthesized results.
Reporting bias assessment	14	Describe any methods used to assess risk of bias due to missing results in a synthesis (arising from reporting biases).
Certainty assessment	15	Describe any methods used to assess certainty (or confidence) in the body of evidence for an outcome.
**RESULTS**		
Study selection	16a	Describe the results of the search and selection process, from the number of records identified in the search to the number of studies included in the review, ideally using a flow diagram.
	16b	Cite studies that might appear to meet the inclusion criteria, but which were excluded, and explain why they were excluded.
Study characteristics	17	Cite each included study and present its characteristics.
Risk of bias in studies	18	Present assessments of risk of bias for each included study.
Results of individual studies	19	For all outcomes, present, for each study: (a) summary statistics for each group (where appropriate) and (b) an effect estimate and its precision (e.g. confidence/credible interval), ideally using structured tables or plots.
Results of syntheses	20a	For each synthesis, briefly summarise the characteristics and risk of bias among contributing studies.
	20b	Present results of all statistical syntheses conducted. If meta-analysis was done, present for each the summary estimate and its precision (e.g. confidence/credible interval) and measures of statistical heterogeneity. If comparing groups, describe the direction of the effect.
	20c	Present results of all investigations of possible causes of heterogeneity among study results.
	20d	Present results of all sensitivity analyses conducted to assess the robustness of the synthesized results.

Section and Topic	Item #	Checklist item
Reporting biases	21	Present assessments of risk of bias due to missing results (arising from reporting biases) for each synthesis assessed.
Certainty of evidence	22	Present assessments of certainty (or confidence) in the body of evidence for each outcome assessed.
**DISCUSSION**		
Discussion	23a	Provide a general interpretation of the results in the context of other evidence.
	23b	Discuss any limitations of the evidence included in the review.
	23c	Discuss any limitations of the review processes used.
	23d	Discuss implications of the results for practice, policy, and future research.
**OTHER INFORMATION**		
Registration and protocol	24a	Provide registration information for the review, including register name and registration number, or state that the review was not registered.
	24b	Indicate where the review protocol can be accessed, or state that a protocol was not prepared.
	24c	Describe and explain any amendments to information provided at registration or in the protocol.
Support	25	Describe sources of financial or non-financial support for the review, and the role of the funders or sponsors in the review.
Competing interests	26	Declare any competing interests of review authors.
Availability of data, code and other materials	27	Report which of the following are publicly available and where they can be found: template data collection forms; data extracted from included studies; data used for all analyses; analytic code; any other materials used in the review.

From: Page MJ, McKenzie JE, Bossuyt PM, Boutron I, Hoffmann TC, Mulrow CD, et al. The PRISMA 2020 statement: an updated guideline for reporting systematic reviews. BMJ 2021;372:n71. doi: 10.1136/bmj.n71

For more information, visit: http://www.prisma-statement.org/

Supplementary Table 2. Study quality

	Items														
Study	1	2	3	4	5	6	7	8	9	10	11	12	13	14	Score
Chen	Y	Y	N	N	Y	Y	N	Y	Y	N	Y	Y	N	Y	8
Molavi	Y	Y	N	Y	Y	Y	N	Y	Y	N	N	Y	Y	N	8

Chu	Y	N	Y	Y	Y	Y	N	Y	Y	Y	Y	Y	Y	Y	12
Castro	Y	Y	Y	N	Y	Y	N	Y	Y	Y	N	Y	Y	Y	10
Akhter	N	Y	Y	Y	Y	Y	N	Y	Y	N	Y	N	N	Y	8
Casale	Y	Y	Y	N	Y	Y	N	Y	Y	N	Y	N	Y	Y	9
Dempsey	Y	Y	Y	Y	Y	N	N	Y	Y	N	Y	Y	Y	Y	10
Annoni	Y	N	Y	Y	Y	Y	N	Y	Y	Y	Y	N	N	N	11
Zorbaz	Y	N	Y	Y	Y	N	N	Y	Y	Y	Y	N	N	Y	9
Feng	Y	Y	N	Y	Y	N	N	Y	Y	Y	Y	Y	N	N	8
Apaolaza	Y	Y	N	Y	Y	Y	N	Y	Y	N	Y	Y	Y	Y	9
de Bérai	Y	N	Y	Y	Y	Y	N	Y	Y	N	Y	Y	N	Y	9
D.Chen	Y	Y	N	Y	Y	Y	N	Y	Y	Y	N	Y	N	Y	9
Darcin	Y	N	N	N	Y	N	N	Y	Y	Y	Y	Y	N	Y	7
Kong	Y	Y	N	N	Y	Y	N	Y	Y	N	Y	Y	Y	Y	8
Liu	Y	N	Y	Y	Y	Y	N	Y	Y	N	N	Y	N	Y	8
Yan	Y	Y	Y	Y	Y	Y	N	Y	Y	Y	Y	Y	N	Y	12
Mazalin	Y	Y	Y	Y	Y	Y	N	Y	Y	Y	Y	Y	N	Y	12
B. Dong	Y	Y	Y	Y	Y	N	N	Y	Y	Y	Y	N	Y	Y	11
Sertbaş	Y	N	N	N	Y	Y	N	Y	Y	Y	Y	N	Y	Y	8
Peterka-Bonetta	Y	N	Y	Y	Y	N	N	Y	Y	Y	N	N	Y	Y	8
Liu	Y	Y	Y	Y	Y	Y	N	Y	Y	N	N	N	Y	Y	11
Jiang	Y	N	N	Y	Y	Y	N	Y	Y	Y	N	Y	N	Y	7
Li	Y	Y	Y	N	Y	Y	N	Y	Y	Y	Y	Y	Y	Y	10
Zhou	Y	N	N	N	N	Y	N	Y	Y	N	Y	N	N	Y	8
Qin	Y	N	N	Y	Y	Y	N	Y	Y	N	Y	Y	Y	Y	11
Andreou	Y	Y	Y	N	N	N	N	Y	Y	Y	N	Y	Y	N	9
Chen	Y	Y	Y	Y	Y	Y	N	Y	Y	N	Y	Y	Y	Y	9
Li	Y	N	N	Y	Y	Y	N	Y	Y	N	Y	Y	Y	Y	9
Wan	Y	N	Y	N	Y	Y	N	Y	Y	Y	Y	Y	Y	Y	10
Zhou	Y	Y	N	Y	N	N	N	Y	Y	N	Y	N	N	Y	8

Gao	N	Y	Y	Y	Y	Y	N	Y	Y	Y	Y	N	N	Y	11
Wang	Y	N	Y	Y	Y	N	N	Y	Y	Y	Y	N	Y	Y	10
Teng	Y	Y	Y	N	Y	N	N	Y	Y	Y	Y	Y	N	Y	10
Zhang	Y	N	Y	Y	Y	Y	N	Y	Y	Y	Y	N	Y	Y	11
Xiang	Y	N	Y	Y	Y	N	N	Y	Y	N	Y	Y	Y	Y	9
Wang	Y	Y	Y	Y	Y	N	N	Y	Y	Y	Y	Y	N	N	9
Zhang	Y	Y	Y	N	Y	Y	N	Y	Y	Y	Y	Y	Y	Y	10
Chen	N	Y	Y	Y	Y	Y	N	Y	Y	Y	Y	N	N	Y	11

Note. Item numbers 1 to 14 in the table indicate the following contents:

1. Was the research question or objective in this study clearly stated?
2. Was the study population clearly specified and defined?
3. Were the study participants selected in an unbiased way?
4. Was the exposure or condition measured in a valid and reliable way?
5. Was the outcome or condition measured in a valid and reliable way?
6. Were the statistical analyses appropriate for the study design and type of data collected?
7. Were potential confounding variables identified and accounted for in the analysis?
8. Were the study participants followed up for the appropriate amount of time?
9. Was the loss to follow-up or drop-out rate for the study acceptable?
10. Were the study participants enrolled in the study in a timely and efficient manner?
11. Were the study participants managed in a consistent way throughout the study?
12. Were the sources of funding or support for the study reported?
13. Was the study approved by an ethics committee or institutional review board?
14. Were the results of the study clearly presented and reported?

Supplementary Table 3. Literature Search strategies

**Database**	**Search strategies**
PsycINFO	(Internet addict* OR Online addict* OR Excess* Internet OR Problem* Internet OR Patholog* Internet OR Excess* computer OR Internet game* addict* OR Internet game* disorder OR "IAD" OR "PIU" OR "IGD" OR "cyberaddiction" OR "internet overuse") AND ("Social anxiety" OR "interaction anxiousness" OR "interaction anxiety" OR social avoidance distress)
Web of Science	(TS=Internet addict* OR TS=Online addict* OR TS=Excess* Internet OR TS=Problem* Internet OR TS=Patholog* Internet OR TS=Excess* computer OR TS=Internet game* addict* OR TS=Internet game* disorder OR TS="IAD" OR TS="PIU" OR TS="IGD" OR TS="cyberaddiction" OR TS="internet overuse") AND

	(TS="Social anxiety" OR TS="interaction anxiousness" OR TS="interaction anxiety" OR TS=social avoidance distress)
PubMed	(Internet addict* OR Online addict* OR Excess* Internet OR Problem* Internet OR Patholog* Internet OR Excess* computer OR Internet game* addict* OR Internet game* disorder OR "IAD" OR "PIU" OR "IGD" OR "cyberaddiction" OR "internet overuse") AND ("Social anxiety" OR "interaction anxiousness" OR "interaction anxiety" OR social avoidance distress)
Scopus	(Internet addiction OR Online addiction OR excessive Internet OR "IAD" OR cyberaddiction OR internet overuse) AND (Social anxiety OR interaction anxiousness OR interaction anxiety)
CNKI(in Chinese)	(TKA=网络成瘾 OR TKA=互联网成瘾障碍 OR TKA=网络滥用 OR TKA=网络过度使用 OR TKA=网络病理性使用 OR TKA=网络强迫性使用 OR TKA=网络强迫症 OR TKA=网络沉迷 OR TKA=网络依赖 OR TKA =网络依恋) AND (TKA=社交焦虑 OR TKA=社交焦虑 OR TKA=交往焦虑 OR TKA=互动焦虑 OR TKA=社会焦虑)
VIP(in Chinese)	(U=网络成瘾 OR U=互联网成瘾障碍 OR U=网络滥用 OR U=网络过度使用 OR U=网络病理性使用 OR U=网络强迫性使用 OR U=网络强迫症 OR U=网络沉迷 OR U=网络依赖 OR U =网络依恋) AND (U=社交焦虑 OR U=社交焦虑 OR U=交往焦虑 OR U=互动焦虑 OR U=社会焦虑)

Supplementary Table 4. Meta-regression of publication year

Table 1 Meta-regression of publication year

_ES	*Coefficient*	*Std. Err.*	*t*	*P*	95% CI
year	0.014	0.006	2.09	0.044	[0.004, 0.281]
_cons	-28.397	13.7567	-2.06	0.046	[-56.271, -0.523]

Abbreviations: year, publication year.

Supplementary Figure 1. Forest plot of Subgroups

Note: 1=Male>Female, 2=Female>Male in the Forest plot of gender

Figure1.1Forest plot of Measurement of PIU

Figure1.2 Forest plot of Measurement of cultural context

Figure1.3 Forest plot of Measurement of gender

Figure1.4 Forest plot of Measurement of social anxiety

Figure1.5 Forest plot of Developmental Level

Figure1.6 Forest plot of database

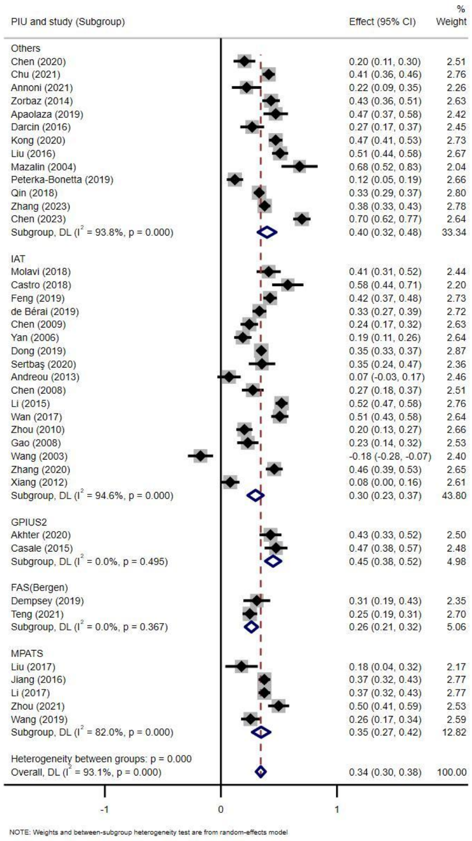

Figure1.1

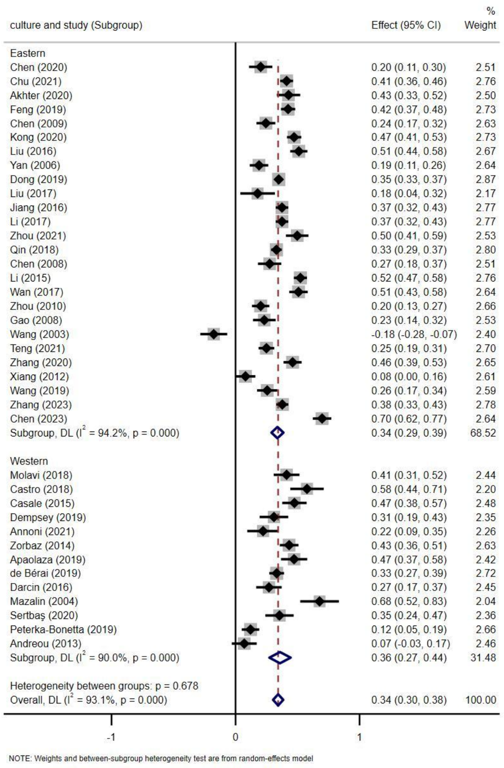

Figure1.2

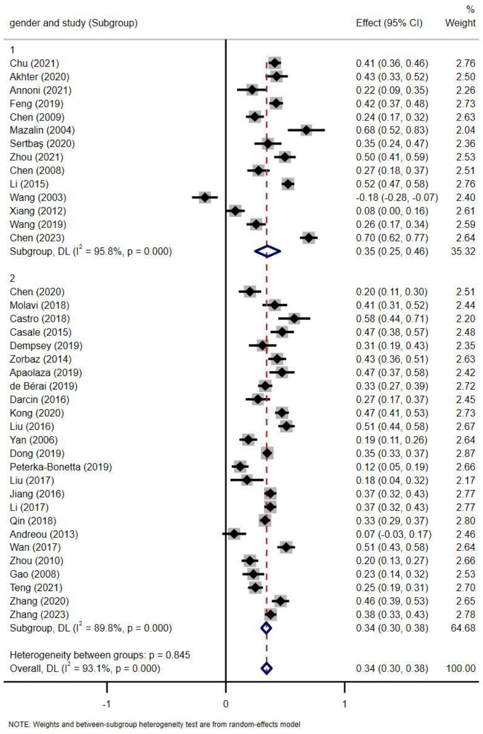

Figure1.3

(Figure Legend: '1' represents studies where the number of male participants surpasses that of female participants, while '2' denotes studies where female participants outnumber male participants.)

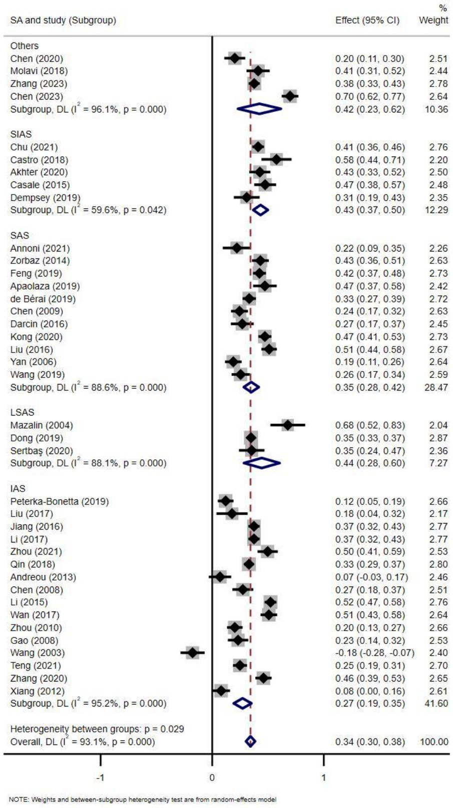

Figure1.4

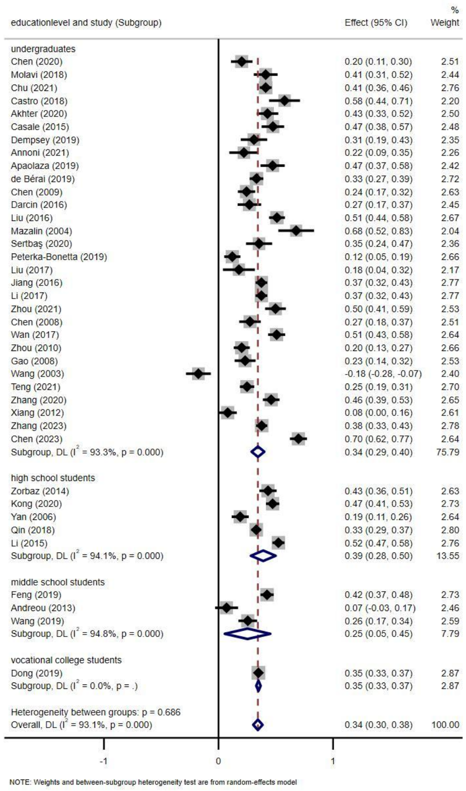

Figure1.5

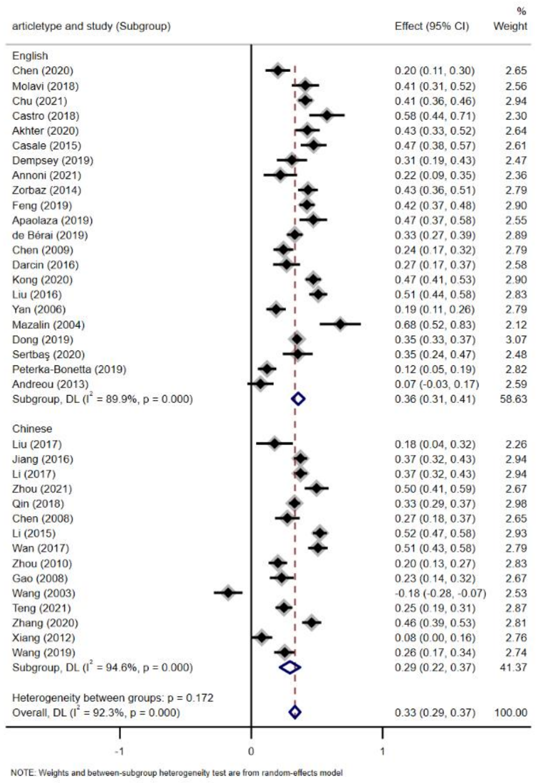

Figure1.6

Figure Legend: 'English' denotes articles obtained from English-language databases, and 'Chinese' signifies articles sourced from Chinese-language databases.

Supplementary Figure 2. sensitivity analysis

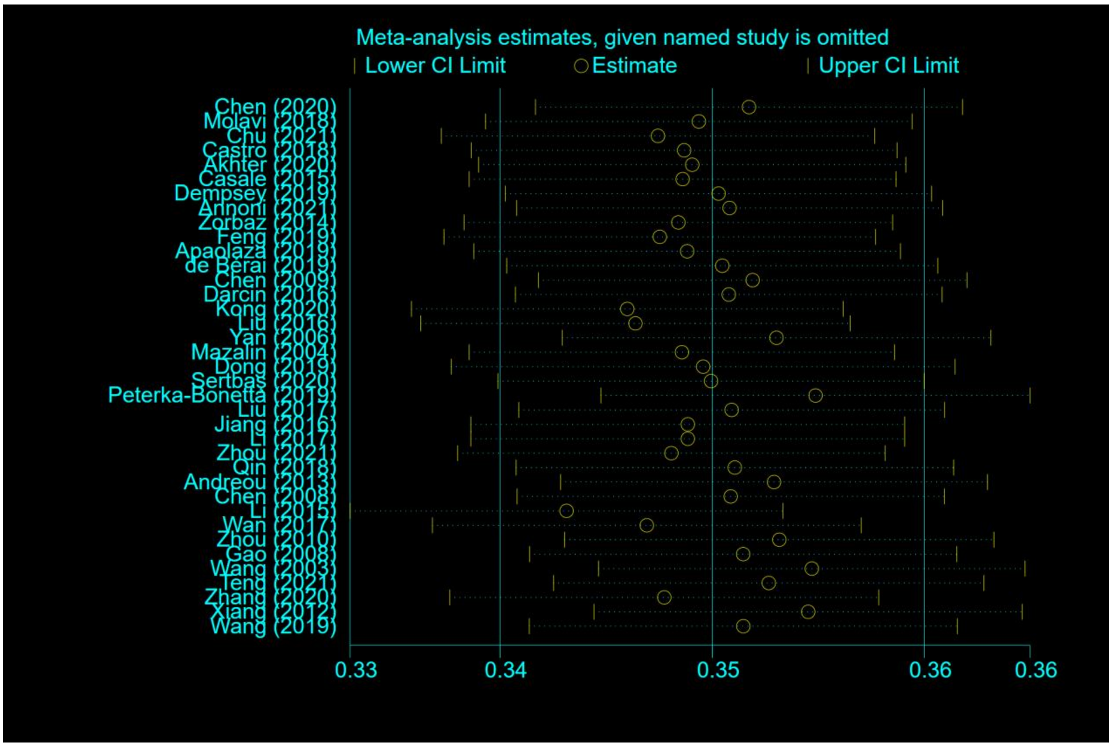

Supplement: Supplementary file 1 [file Data_Sheet_1.pdf]
